# Characterization of Chemically Activated Pyrolytic Carbon Black Derived from Waste Tires as a Candidate for Nanomaterial Precursor

**DOI:** 10.3390/nano10112213

**Published:** 2020-11-06

**Authors:** Reyna Berenice González-González, Lucy T. González, Sigfrido Iglesias-González, Everardo González-González, Sergio O. Martinez-Chapa, Marc Madou, Mario Moisés Alvarez, Alberto Mendoza

**Affiliations:** 1 Tecnologico de Monterrey, Escuela de Ingeniería y Ciencias, Ave. Eugenio Garza Sada 2501, Monterrey 64849, N.L., Mexico; A00818415@itesm.mx (R.B.G.-G.); lucy.gonzalez@tec.mx (L.T.G.);sigfrido@tec.mx (S.I.-G.); A00817961@itesm.mx (E.G.-G.); smart@tec.mx (S.O.M.-C.);mario.alvarez@tec.mx (M.M.A.); 2 Department of Mechanical and Aerospace Engineering, University of California, Irvine, CA 92697, USA; mmadou@uci.edu

**Keywords:** particle characterization, pyrolysis, carbon dots, valorization, chemical activation

## Abstract

Pyrolysis is a feasible solution for environmental problems related to the inadequate disposal of waste tires, as it leads to the recovery of pyrolytic products such as carbon black, liquid fuels and gases. The characteristics of pyrolytic carbon black can be enhanced through chemical activation in order to produce the required properties for its application. In the search to make the waste tire pyrolysis process profitable, new applications of the pyrolytic solid products have been explored, such as for the fabrication of energy-storage devices and precursor in the synthesis of nanomaterials. In this study, waste tires powder was chemically activated using acid (H_2_SO_4_) and/or alkali (KOH) to recover pyrolytic carbon black with different characteristics. H_2_SO_4_ removed surface impurities more thoroughly, improving the carbon black’s surface area, while KOH increased its oxygen content, which improved the carbon black’s stability in water suspension. Pyrolytic carbon black was fully characterized by elemental analysis, inductively coupled plasma–optical emission spectrometry (ICP-OES), Fourier transform infrared spectroscopy (FTIR), Raman spectroscopy, X-ray diffraction (XRD), N_2_ adsorption/desorption, scanning electron microscopy–energy-dispersive X-ray spectroscopy (SEM-EDS), dynamic light scattering (DLS), and ζ potential measurement. In addition, the pyrolytic carbon black was used to explore its feasibility as a precursor for the synthesis of carbon dots; synthesized carbon dots were analyzed preliminarily by SEM and with a fluorescence microplate reader, revealing differences in their morphology and fluorescence intensity. The results presented in this study demonstrate the effect of the activating agent on pyrolytic carbon black from waste tires and provide evidence of the feasibility of using waste tires for the synthesis of nanomaterials such as carbon dots.

## 1. Introduction

The environmental problems related to the inadequate disposal of 1.2 billion waste tires annually worldwide, in addition to another four billion already accumulated in landfills and stockpiles, have led to an urgent need for their proper utilization and valorization [1]. In this context, circular economy approaches have become increasingly relevant, particularly those that account for the recovery and valorization of materials during the processing of waste [2,3].

During tire manufacturing, rubber compounds are processed. Additives, such as sulfur and other chemicals, are added to render a crosslinked structure that enhances some physical properties of the final product, such as durability, wear resistance, abrasion and weathering resistance, and mechanical properties [4,5,6]. These properties make them desirable as tires, but also complicate their disposal and cause environmental pollution problems if they are not correctly handled and disposed of [4]. Nonetheless, the valorization of tires is appealing.

Different thermochemical techniques have been investigated for conversion of waste tires into valuable products, such as pyrolysis and wet pyrolysis (or hydrothermal carbonization). Pyrolysis involves heating a material with low moisture content in an inert atmosphere at elevated temperatures (>400 °C) [7,8], while wet pyrolysis consists on the carbonization of the feedstock in hot water medium (180–250 °C, 10–40 bar). In the latter, there is no limitation on the material moisture content [7,9] and the recovered solid presents a higher oxidation degree [10]. Waste tire characteristics, such as their low moisture and ash content combined with their high heating value and volatile matter content make pyrolysis more suitable for its valorization [8]. In this manner, pyrolysis represents a feasible solution to the problem of the inadequate disposal of tires since it enables the recovery of liquid fuels, gases and a solid product [8,11,12]. The solid product consists of a mixture of carbon black (~90%) and inorganic material (~10%) used during tire manufacturing [5]; therefore, is commonly referred as pyrolytic carbon black [13].

Although all the recovered pyrolytic products (solid, liquid, and gas) have well-known applications [11], it has been reported that the profitability of waste tire pyrolysis relies on the final solid fraction application [13,14,15,16,17]. Therefore, a significant amount of research has focused on enhancing the quality of pyrolytic carbon black in order to achieve commercial applications. One of the processes used for this purpose is activation, either physical or chemical [18,19]. Physical activation, a simple and low-cost process, allows the use of agents such as steam and CO_2_ [20]. However, it has been found that physical activation was not as effective as chemical activation developing microporosity [21]. Chemical activation consists of the impregnation of waste tire powder with a chemical agent prior to carbonization [20]. A wide variety of chemical agents have been used with the aim of activating waste tire powders, including H_2_SO_4_ [22,23], H_2_O_2_ [24], H_3_PO_4_ [24,25], HCl [23], and KOH [18,25,26,27,28,29]. The nature of the activating agent has a strong effect on carbon black characteristics [30]. Although chemical activation is a frequently performed process, the chemical reactions involved are not completely understood [20], which generates uncertainty in predicting the properties of the pyrolytic products.

Applications of pyrolytic carbon from waste tires have centered around its use as reinforcement on the rubber industry [31] and as an adsorbent [32]. However, some studies have explored energy-storage applications [22,28,29,33], and some other studies suggest the use of pyrolytic carbon black from tires as a precursor for nanomaterials for sensors and batteries, among other applications [34,35]. In particular, carbon dots, nanomaterials with a size below 10 nm and a quasi-spherical shape, have assumed relevance in different areas due to their fluorescence and biocompatibility properties [36,37,38,39]. However, synthesizing carbon dots through cheap and environmentally friendly methods remains a challenge [40]. Accordingly, the choice of precursors for carbon dots is significant. In this respect, fabrication from waste is an excellent option [41] since the process converts a material with no economic value into a high-value material.

The use of ordinary carbon black from waste tires to synthesize carbon dots has been attempted. Mohammad et al. converted tires into carbon dots of 2–3 nm through an hydrothermal process for their use as photosensitizers [42], while other studies have suggested the use of carbon dots from tires in fluorescence imaging and optoelectronic devices [43]. These recent works have focused on the characterization and applications of the carbon dots produced. Accordingly, the characterization of the carbon black used as precursor is receiving less attention. The characterization of pyrolytic carbon black as a precursor for carbon dots is important because the properties of carbon black are not standardized—they depend on the conditions of the pyrolysis process [13] in the same way that carbon dots’ characteristics strongly depend on their precursor’s nature [44].

In addition, the use of carbon black with modified porosity and surface chemistry by chemical activations might have an effect on the carbon dots’ properties. In this context, the objective of this study is to recover and characterize pyrolytic carbon black from waste tires with modified characteristics by chemical activations as a candidate for its use as a precursor for the production of carbon dots. Carbon black was recovered from the waste tire pyrolysis process using two different activating agents (H_2_SO_4_ and KOH). The effect caused on carbon black’s properties—such as its surface structure, chemistry, and stability in aqueous suspension—by the activating agent are analyzed in detail. In addition, carbon dots were synthesized using the pyrolytic carbon black and a preliminary characterization was conducted (microscopy and fluorescence), demonstrating its presence and the feasibility of using waste tires as a precursor for this type of nanomaterial.

## 2. Materials and Methods

### 2.1. Carbon Black and Carbon Dots Synthesis

**Figure 1:**
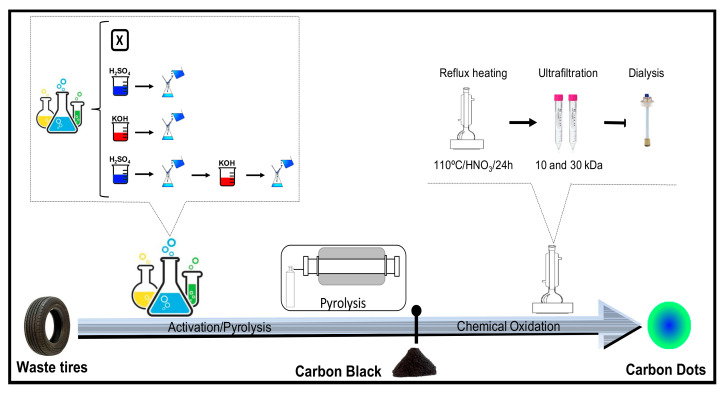
Methodology followed for the recovery of pyrolytic carbon black from waste tires and the synthesis of carbon dots.

Four different processes for the recovery of pyrolytic carbon black from waste tire powder were investigated: (1) pyrolysis, (2) H_2_SO_4_ activation and pyrolysis, (3) pyrolysis and KOH activation, and (4) H_2_SO_4_ activation, pyrolysis, and KOH activation (Figure 1). The parameters of the pyrolysis, the activation process and the chemical oxidation were fixed for all the four processes with the aim of observing only the effect of the activating agent used (H_2_SO_4_ or KOH). These activating agents were selected because among the large variety of chemical agents, KOH is widely used for developing a porous network in carbon materials with a high surface area [45], while H_2_SO_4_ has been found to be effective in generating a controlled microporosity [22].

Waste tire powder with a particle size of 75–125 µm was obtained from LT LeHigh Technologies (Tucker, GE, USA). Activation using H_2_SO_4_ was conducted following an adapted version of the methodology suggested by Naskar et al. [22]: waste tire powder was impregnated with concentrated H_2_SO_4_ at 80 °C under continuous stirring using an H_2_SO_4_/tire weight ratio of 4. The wet material was then filtered under a vacuum, washed, and dried. Similarly, for KOH activation, waste tire powder was impregnated with a KOH/tire weight ratio of 4 over 14 h at 80 °C under continuous stirring before being filtered and dried. Five mL of water were added per gram of KOH for the dilution of previous impregnation. The weight ratio (activating agent/tire) of 4 was selected based on research reporting it as the optimal ratio for enhancing properties like surface area and pore volume [18,25].

The pyrolysis process was performed in a Lindberg blue M tubular muffle (Thermo Scientific^TM^, Watertown, WI, USA) under atmospheric pressure. It was heated at a rate of 10 °C/min until reaching a maximum temperature of 950 °C with a zero holding time to avoid an enhanced carbon gasification that could remove carbon atoms and widen micropores [25]. A temperature of at least 500 °C is required during waste tire pyrolysis to ensure a complete thermal decomposition [4]; however, we use a higher temperature with the aim of causing a turbostratic structure formation, since higher temperatures are used to align graphitic layers [46]. During the whole procedure, a nitrogen flow of 200 mL/min (21 °C, 1 atm) was supplied. The carbon black obtained was collected, characterized, and used as a precursor in carbon dots synthesis through chemical oxidation.

The chemical oxidation procedure used on the carbon black is based on an adapted version of the method followed by Dong et al. for synthesizing carbon dots from activated carbon precursors [47]. Reflux heating was performed at 110 °C with 6M HNO_3_ over 24 h with continuous stirring; NaOH was then used for neutralization purposes. Finally, purification was performed by ultrafiltration and dialysis for 15 days using a 10 mL Spectra-Por^®^ Float-A-Lyzer^®^ G2 (Spectrum Labs, Rancho Dominguez, CA, USA) with a 3.5–5 kDa membrane. In the following sections of the manuscript, the products obtained are identified with “CB” for carbon black, followed by the activating agent used. For example, carbon black from process (4) (i.e., H_2_SO_4_ activation, followed by pyrolysis and KOH activation) is identified as CB.H_2_SO_4_ + KOH.

### 2.2. Waste Tire Powder Characterization

Waste tire powder was characterized by proximal and elemental analyses. Moisture content was determined by ASTM D3173 [48], ash content by ASTM D3174 [49], volatile matter by ASTM D3175 [50], and fixed carbon was calculated as indicated in ASTM D3172 [51]. The elemental analysis was performed in duplicate using an Organic Elemental CHNS-O Analyzer Flash 2000 (Thermo Scientific^TM^, Waltham, MA, USA) equipped with two chromatographic columns and a thermal conductivity detector. The CHNS determination was performed at 900 °C, while oxygen determination occurred at 1060 °C.

### 2.3. Pyrolytic Carbon Black Characterization and Preliminary Analysis of Carbon Dots

The chemical composition, surface morphology, structural information, particle size, and dispersion stability of each pyrolytic carbon black sample were analyzed in detail in order to understand the effect of the activating agent on the carbon’s characteristics. The surface area, presence of micropores, carbon and oxygen content, surface charge, and particle size of the pyrolytic carbon are some of the relevant characteristics to consider for its valorization, either as activated carbon used as adsorbent [20,52] or nanomaterial precursor.

The carbon’s chemical composition was analyzed using the Organic Elemental CHNS-O Analyzer Flash 2000 (Thermo Scientific^TM^, Waltham, MA, USA) in duplicate, and the determination of metallic elements was performed after a microwave-acid digestion with concentrated nitric acid (14 mol/L). The analysis was performed using a Thermo Scientific^TM^ ICP-OES model 6000 (Waltham, MA, USA) with a radio frequency power of 1150 W, an auxiliary gas flow rate of 0.5 L/min, and a nebulizer flow rate of 0.7 L/min. Elements measured (Cd, Cr, Fe, Ni, Pb, and Zn) were selected based on previous bibliographic research on metals presented in tires [53].

The surface morphology of pyrolytic carbon black was evaluated with N_2_ adsorption/desorption isotherms measured at 77 K on an Autosorb IQ TPX Quantachrome gas adsorption analyzer (Boynton Beach, FL, USA). The surface area was calculated using the Brunauer–Emmett–Teller (BET) method [54]. All carbon black samples were outgassed at 250 °C for 12 h before analysis under vacuum conditions. Pore size distribution was calculated using the density functional theory (DFT) method [55].

Structural analysis through Raman spectroscopy was performed using a LABram HR Evolution (Horiba, Jobin Yvon, France) with a 532 nm laser as an excitation source. Raman-scattered light was detected by a Charge Coupled Device (CCD) camera operating at 220 K. An Olympus microscope SLMPN (Tokyo, Japan) 50x/0.35 NA was used to focus the laser on the sample. Raman signals were acquired at 25%, 50%, and 100% of the beam intensity. Band intensity, band position, and area were obtained using a Lorentz curve-fitting procedure on the normalized Raman spectra. Inorganic impurities of a crystalline nature were identified using the X-ray diffraction (XRD) technique. The Panalytical Empyrean X-ray diffractometer (Malvern, UK) was equipped with an ultra-fast X’Celerator detector with a Bragg–Brentano geometry. It operated at 45 kV and 40 mA with a Cu radiation (Kα = 15.405 Å) in the range of 5° to 90°, with a size of 0.033 and a dwell time of 60 s in 2θ. Surface chemistry was studied with a Frontier^TM^ FTIR (Fourier transform infrared) Spectrometer (PerkinElmer, Waltham, MA, USA) with the Universal ATR (attenuated total reflectance) sampling accessory. The measurements were performed in the transmission range of 400 cm^−1^ to 4000 cm^–1^ with 64 scans.

Hydrodynamic diameters were measured by dynamic light scattering (DLS) and ζ potential was measured by phase analysis light scattering (PALS), both using a Nanobrook 90 Plus PALS Brookhaven instrument (Holtsville, NY, USA). Carbon black powder was dispersed in mili-Q water and sonicated for 5 min before analysis. Ten DLS measurements were performed per sample with a set duration of 100 s and an equilibration time of 300 s. For ζ potential, ten measurements per sample were performed, and the Smoluchowski equation was used to convert the electrophoretic mobility of the particles measured by the instrument into the ζ potential.

The surface morphology of the pyrolytic carbon black was analyzed using two scanning electron microscopes (SEM): a JEOL model JSM-6010PLUS/LA (Peabody, MA, USA), which was equipped with a JEOL EX94400T4L11 backscattered electron detector, and a FEI Nova NanoSEM 200 (Hillsboro, OR, USA) with an INCA X-Sight energy-dispersive X-ray spectroscopy (EDS) detector. Both devices were operated at 15 kV in low vacuum mode. Backscattered and secondary electrons were used for the analysis. Samples of carbon black powder was deposited on carbon tape placed on a microscope glass slide.

Transmission electron microscopy (TEM) and fluorescence were used to analyzed carbon dots. TEM images were acquired in a transmission electron microscope Hitachi HT7700 Hitachinaka, Japan) operated at 100 kV. A drop of the carbon dots dispersed in distilled water was deposited on a 300-mesh copper grid with a lacey carbon film. A Synergy HT Biotek Fluorescence microplate reader (Winooski, VT, USA) was used to measure the fluorescence property of the synthetized carbon dots. 200 μL of sample were placed in a 96-well black plate to measure fluorescence in duplicate using water as a control.

## 3. Results and Discussion

### 3.1. Waste Tire Powder Characterization

The waste tire powder used as feedstock was analyzed through proximal and elemental analysis to identify which changes generated in the waste tire powder were due solely to the activation process. Results of non-activated waste tire powder presented in Table 1 are in agreement with those reported typically for steel-free tire powder [15,18,25]. Non-activated and KOH-activated waste tire presented similar results on their proximal analysis (Table 1): moisture, ash, volatile matter, and fixed carbon content were maintained almost equally. Similarly, the KOH-activated waste tire did not show variations from the non-activated waste tire on elemental analysis results except for a slight increase in oxygen content caused by the activation. In contrast, H_2_SO_4_ activation caused variations on the chemical characteristics; specifically, ash and volatile matter content were reduced. The decrease in volatile matter might be due to the fact that during activation, carbon reacts with H_2_SO_4_ to produce carbon dioxide, sulfur dioxide, and water, releasing some of the volatile matter; at the same time, carbon content decreased, as demonstrated in the elemental analysis. On the other hand, the decrease in ash content has been previously studied by some authors [15,56], who reported that H_2_SO_4_ demonstrated good performance as a demineralization agent. This is relevant because a high ash content reduces the quality of carbon black. The decrease in ash and volatile matter content is even higher in the acid-base activation (H_2_SO_4_ and KOH-activated waste tire powder).

Sulfur was observed in all samples because it is used during the vulcanization process in tire manufacturing [17]. However, its content increased with acid-activation due to the type of acid used (H_2_SO_4_). Sulfur presented in the H_2_SO_4_-activated waste tire could be in the form of sulfates (SO_4_^2^^−^) as impurities, which may be responsible for the oxygen content in the proportions shown in Table 1.

### 3.2. Pyrolytic Carbon Black Characterization

#### 3.2.1. Chemical Composition

Recovered pyrolytic carbon black from the four different waste tire powder (non-activated, H_2_SO_4_-activated, KOH-activated and H_2_SO_4_/KOH-activated waste tire) was around 40–47 wt.% of the waste tire. It was characterized to identify the effects caused by the activating agent used. The elemental composition of the solid fraction from waste tires pyrolysis has been previously reported [18] and coincides with results obtained for the CB sample (Table 2). Carbon content increased with acid-activation, which can be attributed to the impurities removed by the acid. Although a sulfur-containing acid was used during acid-activation, causing higher concentrations of sulfur and oxygen in the waste tire powder, the recovered carbon black from this acid-activated tire (CB.H_2_SO_4_) showed a decrease in sulfur and oxygen content compared to any of the carbon black samples. As reported by Susa et al. [57], the distribution of sulfur in the gas fraction is increased at higher temperatures, reducing its content in the liquid and solid fractions. We suggest that sulfates presented in the acid-activated waste tire were decomposed into SO_2_ during pyrolysis, causing a decrease in sulfur and oxygen content in the solid fraction. Since sulfates are not present in the KOH-activated waste tire powder, there is no decomposition of sulfur compounds. Therefore, when KOH activation is involved, sulfur content is maintained, and oxygen is highly increased. This, in turn, reduces the percentage of carbon. On the other hand, CB.H_2_SO_4_ + KOH presented a much higher oxygen content than CB.KOH, possibly due to the increased surface area produced during previous H_2_SO_4_ activation. A larger surface area provides better KOH-tire contact, enhancing its activation process and thereby increasing the oxygen content.

As tires are composed of a complex mixture of materials, including metallic elements, the metal composition was determined. All the evaluated metals were present in the carbon black samples, and their concentration decreased with activation (Table 2). The metal concentration reduction is higher with H_2_SO_4_ activation, suggesting that H_2_SO_4_ is a better agent for impurity removal. This result coincides with Liu and Bi [58], who tested the performance of four different acids for the removal of inorganic compounds in biomasses and concluded that H_2_SO_4_ was the most effective agent. López et al. [15] evaluated the concentration of inorganic species before and after demineralization of a tire-derived char using different combination of reagents. The maximum removal of metallic elements was 73.2% with HNO_3_/H_2_O treatment, followed by 69.6% with HNO_3_/H_2_SO_4_. In this study, the maximum removal of metallic elements was obtained with the H_2_SO_4_-activation and H_2_SO_4_ + KOH-activation with values of 94.4% and 99.3% respectively. Although H_2_SO_4_ activation was more effective, KOH activation showed a reasonable metal removal capacity (77.6%), which was also achieved by Mukherjee and Borthakur [59].

#### 3.2.2. Surface Morphology

**Figure 2:**
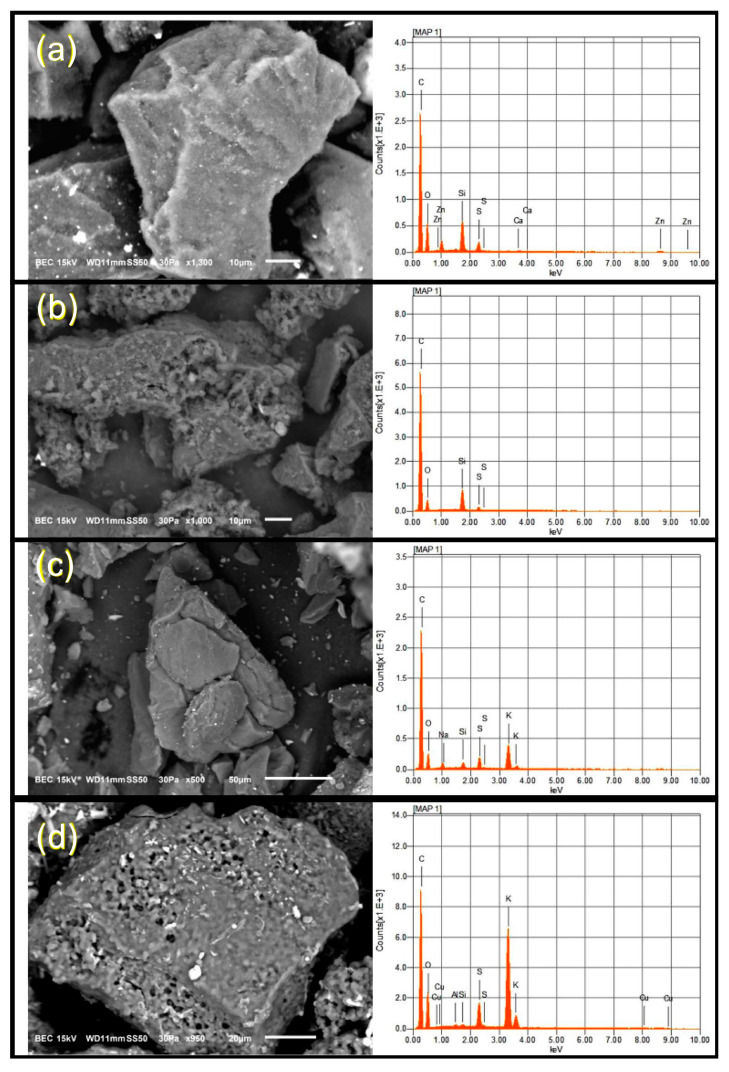
Scanning electron microscopy (SEM) images from (**a**) CB, (**b**) CB.H_2_SO_4_, (**c**) CB.KOH and (**d**) CB.H_2_SO_4_ + KOH. The right column shows the energy-dispersive X-ray spectroscopy (EDS) results from each carbon black sample.

Figure 2 shows some differences on the surface morphology of the carbon black samples. SEM images of CB (Figure 2a) presented an intact external surface, CB.H_2_SO_4_ (Figure 2b) and CB.H_2_SO_4_ + KOH (Figure 2d) showed a micropore formation, while CB.KOH (Figure 2c) has a less porous surface with higher impurity presence, as can be further demonstrated by EDS. Zinc and sulfur were detected with the EDS technique (Figure 2). Its presence is due to the original composition of the tires, and it is consistent with ICP-OES results showing high concentrations of zinc. Potassium was observed in CB.KOH and CB.H_2_SO_4_ + KOH. The presence of potassium has been previously reported after KOH activation, even with subsequent washes [60].

Carbon black samples activated by H_2_SO_4_ (CB.H_2_SO_4_ and CB.H_2_SO_4_ + KOH) presented a reduction of impurities, as shown in the elemental analysis and metal content results. This suggests that during acid activation, the impurities could be removed, leading the spaces previously occupied to cause the micropore formation shown in the SEM images.

**Figure 3:**
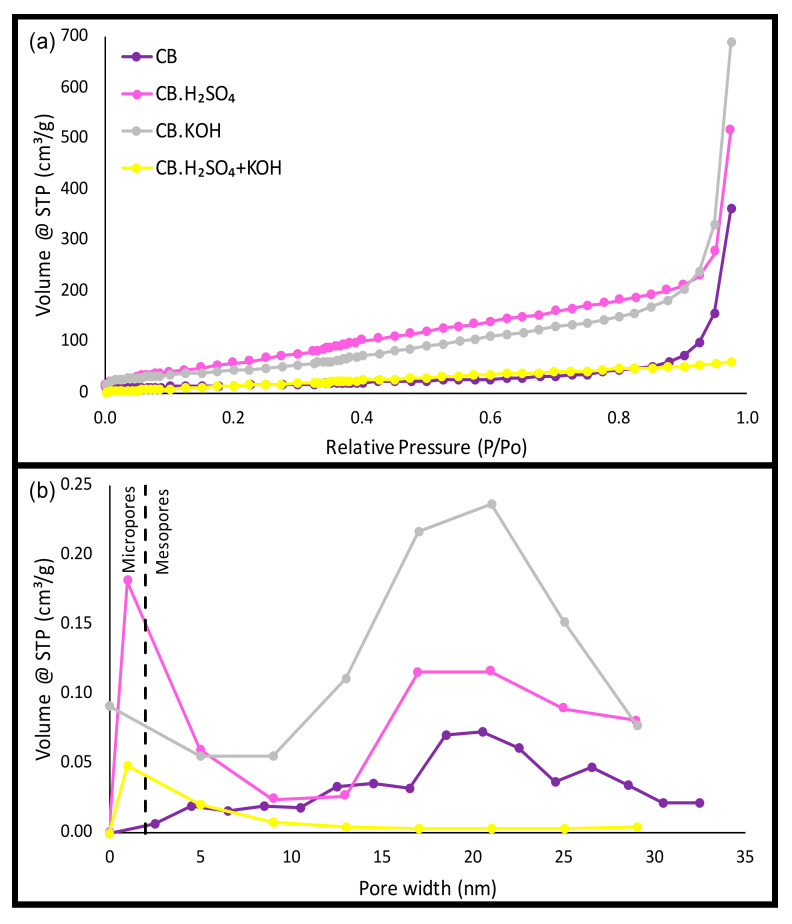
(**a**) N_2_ adsorption/desorption isotherm and (**b**) pore size distribution of carbon black samples.

All carbon black samples exhibited a typical type IV isotherm (Figure 3a) according to the International Union of Pure and Applied Chemistry (IUPAC) classification. This isotherm type indicates a mesoporous structure [61,62], while the observed hysteresis loop type H1 is found in mesoporous materials with ordered three-dimensional pore networks [63]. Of relevance is the prominent microporosity found in H_2_SO_4_-activated samples (CB.H_2_SO_4_ and CB.H_2_SO_4_ + KOH), which is consistent with results reported by Naskar et al. [22], who observed pore widths of less than 2 nm when a chemical pretreatment with H_2_SO_4_ to the tire powder was performed. In contrast, KOH-activation presented mesoporosity [25].

The original tire had a very low surface area value (approximately 0.13 m^2^/g), demonstrating its non-porous structure [64]. CB showed a surface area of 57 m^2^/g, which is a typical value reported for pyrolytic carbon black derived from tires [64,65]. KOH activation produced a higher surface area value (197 m^2^/g). Using the same KOH/tire ratio, Nieto-Márquez et al. [60] obtained char with a surface area of 358 m^2^/g. It should be noted that these authors washed the pyrolytic char with HCl to remove impurities after the heating process. Carbon black from the process with both activation agents (CB.H_2_SO_4_ + KOH) presented a surface area of 82 m^2^/g, a low surface area similar to the CB value. Gómez-Hernández et al. [35] reported occlusion of the pores due to the typical impurities of the tire. Apparently, pores formed during the KOH activation process were filled with impurities, which is also revealed through SEM, EDS, and elemental analysis of CB.KOH and CB.H_2_SO_4_ + KOH samples, resulting in a low surface area value. By contrast, EDS and elemental composition results for CB.H_2_SO_4_ showed a more impurity-free surface. Therefore, this carbon black sample showed the highest surface area value (302 m^2^/g).

Figure 3b illustrates pore size distribution on the carbon black samples. CB and CB.KOH had a greater amount of mesopores (2 to 50 nm), while carbon black samples obtained by processes with H_2_SO_4_ activation (CB.H_2_SO_4_ and CB.H_2_SO_4_ + KOH) presented a prominent microporous structure (pores smaller than 2 nm). This H_2_SO_4_ activation effect on pore size reduction was also observed in previous research comparing H_2_SO_4_ activation with CO_2_ activation on waste tires for energy-storage applications [66].

It was observed that textural properties strongly depend on the activating agent. If activated carbon applications such as adsorbents are desired for carbon black, a washing process with acid or base should be added to increase the surface area value. In this study, carbon black is used to perform a preliminary exploration of its use as precursor for carbon dots synthesis through an oxidation process with HNO_3_. In this context, we assume that an additional washing process would not be required since impurities filling the pores are eliminated during the chemical oxidation process.

#### 3.2.3. Structural Analysis

**Figure 4:**
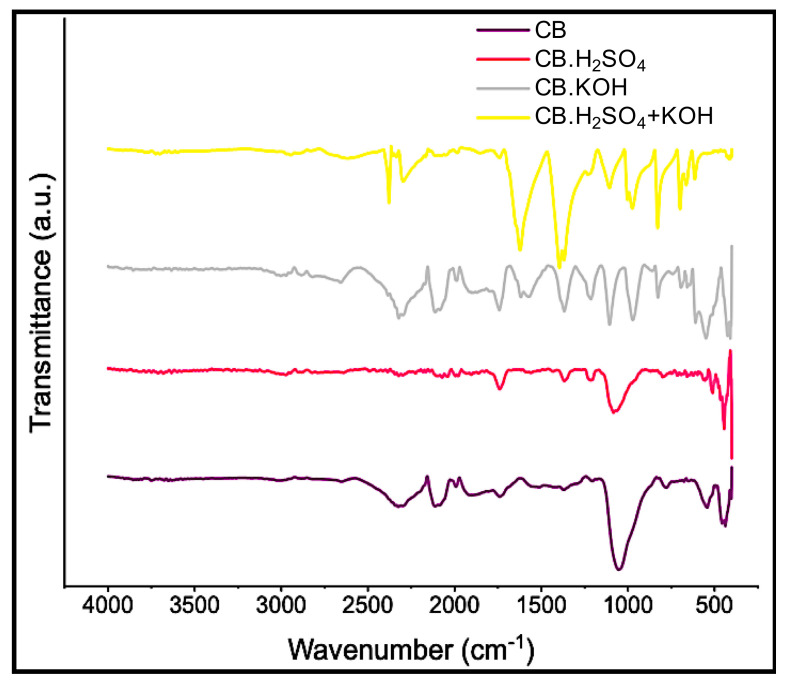
Fourier transform infrared (FTIR) spectra of carbon black samples.

Functional groups and chemical vibrations of the carbon black samples were analyzed by FTIR (Figure 4). In the region from 3000 cm^–1^ to 3200 cm^–1^, which is associated with O–H stretching asymmetric vibrations [35], very weak bands were observed in the activated samples (CB.H_2_SO_4_, CB.KOH and CB.H_2_SO_4_ + KOH), which coincides with the oxygen content observed by other techniques. The band around 2349 cm^–1^ is ascribed to asymmetrical stretching vibrations of CO_2_. Its presence could be associated with the porous nature of carbon black samples [35]. The band in the region from 1650 cm^–1^ to 1860 cm^–1^ is associated with the C=O stretching vibrations corresponding to carbonyl and carboxyl groups [67]; this peak is more pronounced in the carbon black samples with activations. The peak presented in CB.KOH and CB.H_2_SO_4_ + KOH around 1580 cm^–1^ corresponds to aromatic stretching coupled to highly conjugated carbonyl groups C=O [67]. The peak around 1400 cm^–1^ is ascribed either to carboxyl-carbonate structures or to aromatic C=C [67]. Peaks in the region from 800 cm^–1^ to 1200 cm^–1^ correspond to C–C stretching [68]. Bands below 950 cm^–1^ have been assigned to out-of-plane deformation vibrations of C–H groups in aromatic structures [67].

**Figure 5:**
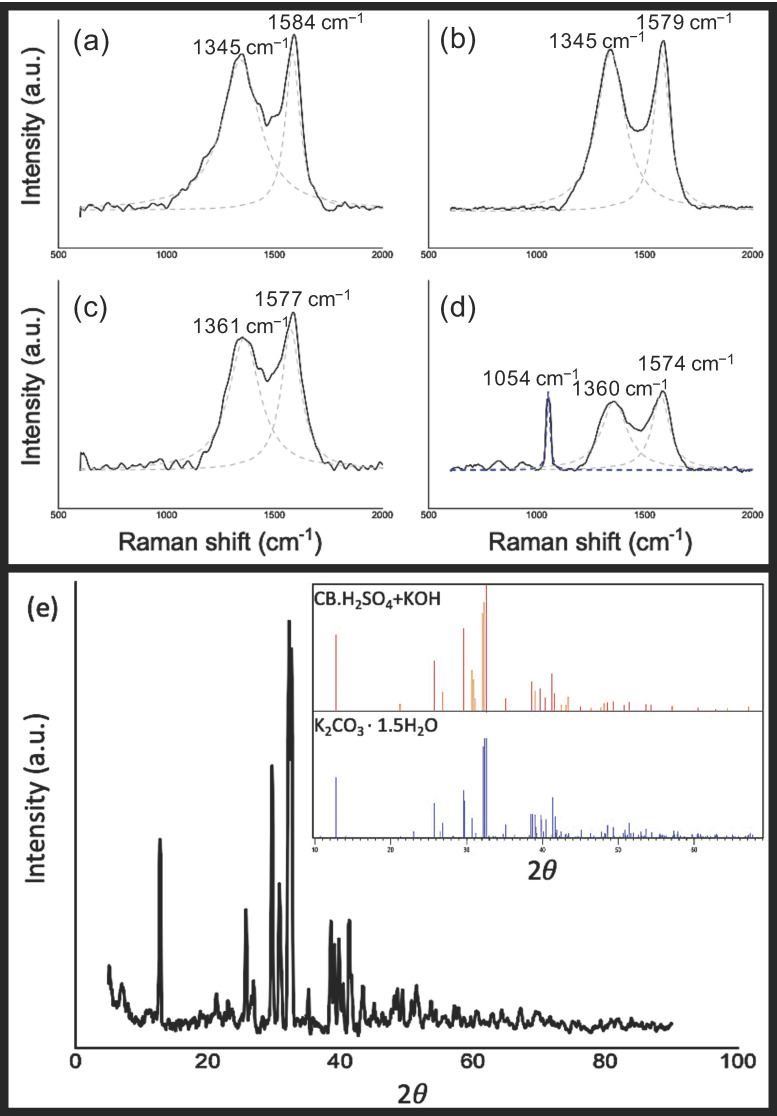
Fitted Raman spectra (black straight line) of (**a**) CB, (**b**) CB.H_2_SO_4_ (**c**) CB.KOH and (**d**) CB.H_2_SO_4_ + KOH. The D and G bands are plotted in a gray dash line (**a**–**d**). (**e**) XRD patterns of CB.H_2_SO_4_ + KOH inset: X-ray diffraction (XRD) pattern of the identified K_2_CO_3_ · 1.5H_2_O, International Centre for Diffraction Data (ICDD) Reference code 98-007-8315).

Carbon black has an amorphous structure; therefore, it was expected that its Raman spectra would present the D band and G band depicted in Figure 5a–d. The D band at approximately 1350 cm^–1^ corresponding to defects on the lattice indicates an amorphous structure and hydrocarbon or aliphatic moieties connected to the graphitic structure [35,69]. The G band at approximately 1580 cm^–1^ corresponding to in-place vibrations demonstrates the presence of small graphitic-planes or microcrystallites. Similar Raman spectroscopy results were reported by Kumar et al. [29], who synthetized turbostratic carbon from waste tires with a I_D_/I_G_ of 1.06. The intensity ratios I_D_/I_G_ from pyrolytic carbon black samples are in the range of 0.93 to 1.01 (Table 3). In addition to the intensity ratio I_D_/I_G_, some researchers [70,71] suggest that G band position and G band width could be considered as graphitization indices as well. The G band positions presented a slight decrease with the activation processes, from 1584 cm^−1^ to 1574 cm^–1^. This effect was also observed with an increasing temperature by Gruber et al. [46], who achieved a G band position similar to graphite with carbon black samples at a temperature of 3000 K. In this study, the G band position of the carbon black samples was the same as for graphite, but it was obtained with a much lower temperature. The I_D_/I_G_ increase presented in CB.H_2_SO_4_ compared to CB could be associated to the decrease on the metal impurities derived from the acid activation. Transition metals are able to catalytically promote the graphitization of carbon [72,73]. Metallic impurities such as Fe, Ni and Zn remain in CB, while its content is considerably decreased in acid-activated carbon black; therefore, we suggest that the metallic impurities removal leads to a lower carbon graphitization. Although CB.H_2_SO_4_ presented a slight I_D_/I_G_ increase, its G band is higher, G band and D band positions are lower, D band width is lower, and G peak is more intense than those presented in CB. Therefore, we conclude that although the I_D_/I_G_ ratio is higher, some degree of graphitization is present, considering the other indices; however, further analysis should be performed to confirm the graphitization of the materials and the effect of the metal impurities.

CB.H_2_SO_4_ + KOH exhibited an additional peak centered at 1054 cm^−1^, which was attributed to dipotassium carbonate sesquihydrate (K_2_CO_3_ · 1.5H_2_O) [74]. These results were verified by the XRD technique as shown in Figure 5e. Its presence is due to the previous KOH activation. Matsukata et al. [75] found that potassium compounds migrate into the carbon lattice with temperatures between 670 and 900 K; at higher temperatures, reactions with carbon occur, causing the reduction of potassium compounds presented on the surface. Although a higher temperature was used in our study, potassium carbonate was still found in CB.H_2_SO_4_ + KOH, while CB.KOH did not show the characteristic peak of potassium carbonate in Raman spectra. This suggests that, apparently, acid activation prior to KOH activation makes it difficult to reduce potassium in the carbon lattice.

#### 3.2.4. Particle Size and Dispersion Stability

**Figure 6:**
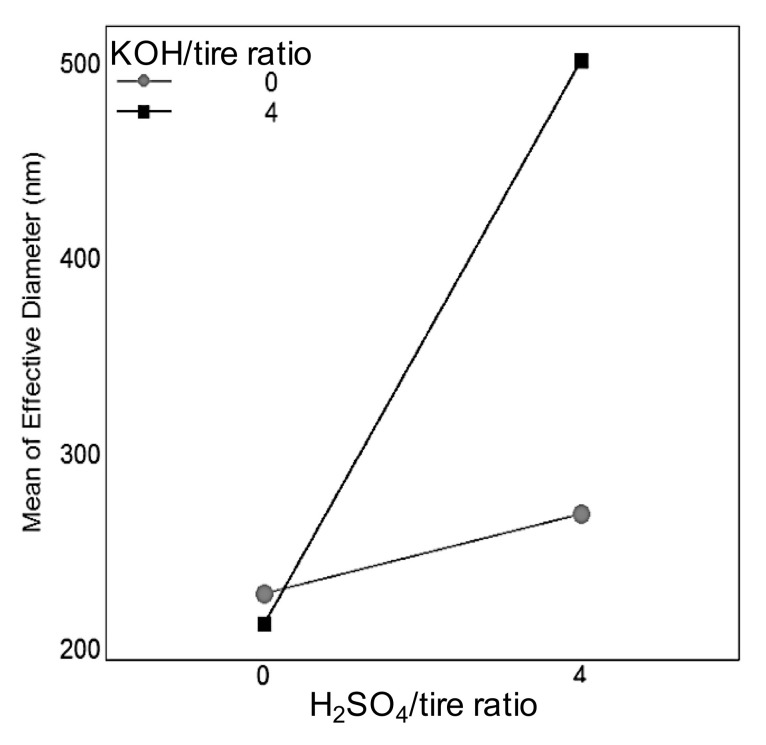
Interaction plot showing the KOH activation effect increasing the effective diameter when H_2_SO_4_ activation is also involved.

**Figure 7:**
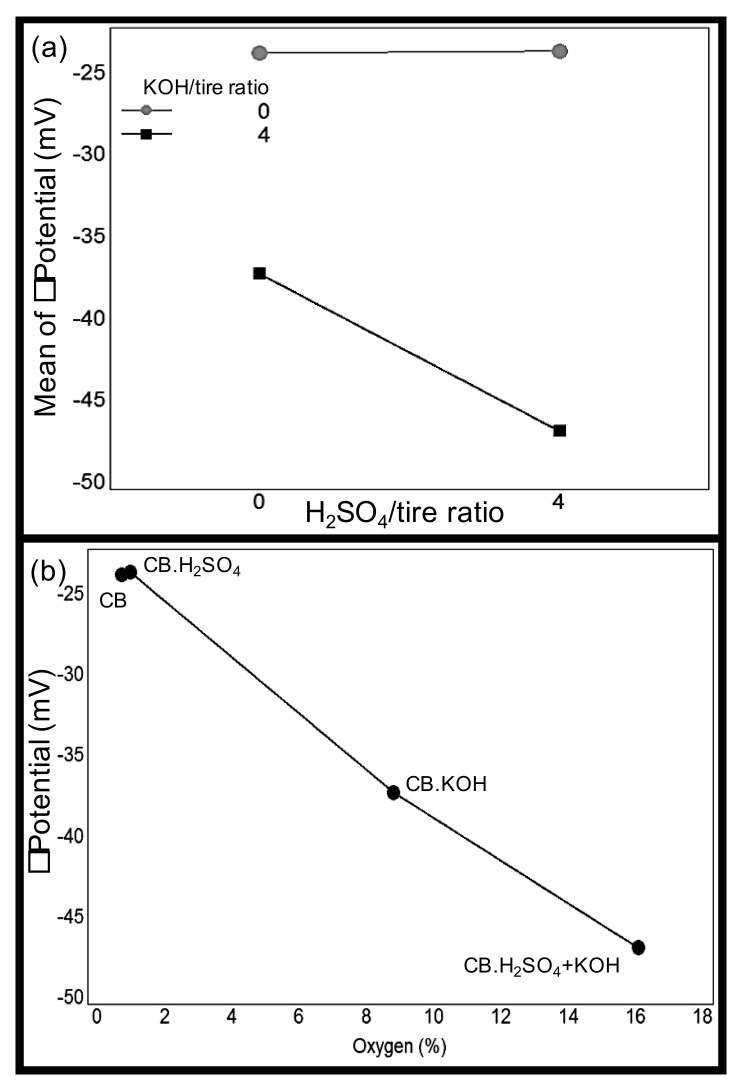
Results of ζ potential analysis. (**a**) Interaction plot showing the KOH activation effect increasing the ζ potential and (**b**) oxygen content effect on the ζ potential of carbon black samples.

The results obtained through the DLS and PALS techniques are presented in Figure 6 and Figure 7. Despite carbon black’s inability to disperse in water [35,76], partially oxidized carbon black nanoparticles are capable of forming stable suspensions [35], so it was possible to measure its particle size distribution and ζ potential in water. Non-activated carbon black and carbon black activated only with one activating agent presented similar particle sizes; CB particles suspended in water have an effective diameter of 228.50 ± 1.19 nm, CB.H_2_SO_4_ of 269.28 ± 9.91 nm, and KOH of 212.77 ± 2.52 nm, while the largest size with a higher standard deviation was obtained in CB.H_2_SO_4_ + KOH with 501.19 ± 141.82 nm.

The interaction plot between the activating agents (how each chemical agent’s effects change according to the presence/absence of the other chemical agent) on the effective diameter of carbon black particles is presented in Figure 6. A significant increase in the effective diameter was observed when both activating agents were involved compared with any of the other options: when the tire was activated only with one agent, the particle size of the recovered carbon black was around 200 nm. However, adding the other activating agent caused an increase above 500 nm in the particle size of the recovered carbon black. Small differences were also observed in the effective diameter of the recovered carbon black when the waste tire was not activated or only one activating agent was used (samples CB, CB.H_2_SO_4_, and CB.KOH), with the highest difference being 56 nm between CB.H_2_SO_4_ and CB.KOH.

The carbon dots synthesis used in this study is based on the methodology of Dong et al. [47], which uses activated carbon powder with a diameter of 200 mesh (around 74 μm) as a precursor. The small size differences presented in the recovered carbon black might not be of interest, since all pyrolytic carbon black samples had a smaller diameter than the precursors used in the reference methodology, and the chemical oxidation process consisted of reducing the size of the precursor until reaching the desired nanoparticle size. No statistical tests were performed since the observed differences are not of interest. The waste tires used in this study had a small particle size (75–125 µm), while recovered carbon black particles were between 213–501 nm. The methodology used requires a carbon precursor of around 74 μm [47]; therefore, the smaller sizes of the carbon black particles might not be necessary for this application. In this context, the use of a tire powder with larger particle size should be further evaluated. On the one hand, the use of a tire powder as fine as that used in this study requires a grinding process that might be too energy intensive [77]. On the other hand, if the particle size is increased, longer residence times for chemical oxidation may be required to achieve the nano-sized carbon dots.

The use of a waste material derived from a highly controlled manufacturing process, such as tires, leads to benefits over other natural or waste nanomaterials precursors. Biomass overcome the disadvantages of the use of not-renewable resources [41,78]; however, their complex composition and processability are obstacles to the synthesis of nanomaterials with homogeneous characteristics [79,80]. Moreover, waste samples frequently are crushed and sieved to separate the desired size fraction before its use [81,82,83,84]. The narrow size distribution of pyrolytic carbon black from tires enables its use as precursor without the requirement of additional sorting processes.

Typically, nanoparticles with ζ potential values between the range of −25 mV to +25 mV have a low degree of stability [85]. Despite the fact that ζ potential values of CB and CB.H_2_SO_4_ are inside the low-stability range, their values are not close to zero. Therefore, although this carbon black sample could eventually aggregate due to Van Der Waal inter-particle attractions, its stability is not very low. On the other hand, CB.KOH and CB.H_2_SO_4_ + KOH presented a value outside the low stability range, indicating that carbon black nanoparticles form a very stable suspension in water, which might be associated with the oxygen content of carbon black after KOH activation. Youssry et al. [86] reported a similar ζ potential value (−25 mV) for carbon black; they explained the negative charge of the carbon surface as a consequence of a possible dissociation of the functional groups with oxygen on the carbon surface.

A two-way analysis of variance (ANOVA) test was performed to evaluate the significance of differences in ζ potential values in relation to the activation process performed. A significant test (*p*-value < 0.001) for the interaction of the two factors (presence of H_2_SO_4_ activation and presence of KOH activation) was obtained; therefore, Tukey’s honestly significant difference (HSD) was performed to identify the significant differences. Tukey’s HSD showed that the ζ potentials of CB and CB.H_2_SO_4_ are not statistically different, while CB.KOH and CB.H_2_SO_4_ + KOH are statistically different from each other and from the other samples (Table 4). Before performing the statistical tests, assumptions of normal distribution and homogeneity of variance were evaluated using a Shapiro–Wilk normality test on the residuals (*p*-value = 0.5529) and a Levene’s test (*p*-value = 0.1801) for homogeneity of variance. Both assumptions were fulfilled by the data.

The interaction plot of the activating agents on ζ potential is presented in Figure 7a. There, the effect of the KOH activation is clear: when there is no KOH activation, the ζ potential remains at the same low absolute value (around 25 mV) regardless of the presence of H_2_SO_4_ activation; however, when KOH activation is involved, the ζ potential absolute value increases, increasing even more when H_2_SO_4_ activation is also added.

Kawaraya et al. [87] found that surface oxidation level is correlated with ζ potential since higher ζ potential absolute values were observed with a higher surface oxidation on carbon black. This oxygen effect was also observed in this study; CB.H_2_SO_4_ + KOH and CB.KOH had a higher oxygen content due to the KOH activation process; accordingly, their ζ potential values were higher (Figure 7b).

### 3.3. Preliminary Analysis of Carbon Dots

**Figure 8:**
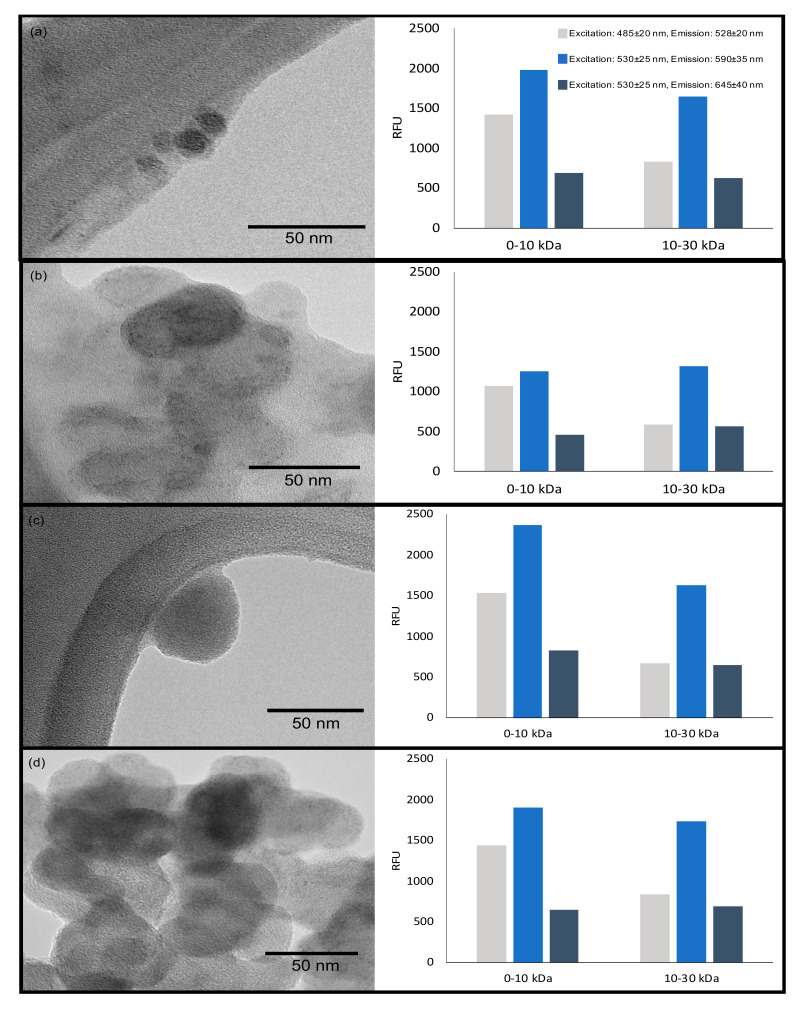
Transmission electron microscopy (TEM) images from (**a**) carbon dots from CB, (**b**) carbon dots from CB.H_2_SO_4_, (**c**) carbon dots from CB.KOH, and (**d**) carbon dots from CB.H_2_SO_4_ + KOH. The bar plots show the mean relative fluorescence units (RFU) at different emission ranges of carbon dots from fraction 0 to 10 kDa and 10 to 30 kDa. Note: Water was used as a control for fluorescence measurements; the maximum difference between the replicas was 101 RFU.

Transmission electron microscopy (TEM) and fluorescence were used to identify synthesized carbon nanoparticles as carbon dots. TEM provided information about their particle size, size distribution and morphology. As can be seen in Figure 8, all the carbon black precursors generated spherically-shaped nanoparticles. Typically, carbon dots are spherical shaped [88,89,90,91], which is in agreement with carbon dots synthesized in this study; however, it has been reported that carbon dots from waste tires synthesized by a physicochemical method was not spherically shaped and monodisperse [43]. The differences in the nanoparticle shape with respect to those reported in literature may be associated to the synthesis method.

Differences in the carbon dots’ sizes were observed depending if the precursor was activated or not: carbon dots from Cd.CB (Figure 8a) presented the smallest particle size (6–8 nm); while carbon dots from activated precursors presented particles in the range of 10–50 nm. The larger sizes observed on carbon dots from activated precursors (CB.H_2_SO_4_, CB.KOH and cd.H_2_SO_4_+KOH; Figure 8b–d) might be associated with an increase in agglomeration caused by the heterogeneity on their particle size and surface chemistry [92]. This agglomeration is also exhibited in TEM images, mainly in carbon dots from CB.H_2_SO_4_ (Figure 8b) and CB.H_2_SO_4_ + KOH (Figure 8d). The observed agglomeration may be caused by the drying process during sample preparation [93] and by the fact that TEM analysis was performed after months of carbon dots synthesis, probably having an effect on carbon dots stability. However, agglomeration and similar size distribution have been reported on carbon dots [94,95].

Fluorescence is one of the main properties of carbon dots. Its importance rests in the fact that many of their applications are based on this property. Additionally, it is used to identify carbon dots in the nanoscale [96]. Differences in the intensity of fluorescence emission were observed: a higher intensity in fluorescence emission at the range of 590 ± 35 nm in all carbon dots samples, and a higher fluorescence emission in the fraction from 0–10 kDa, regardless of the precursor. It has been previously reported that carbon dots from pyrolytic carbon black presented their maximum fluorescence signal at 415 nm [43]. In our study, the maximum fluorescence emission was observed at longer wavelengths, possibly associated with a higher oxidation degree [97,98] caused during the chemical oxidation with HNO_3_.

The comparable fluorescent intensity of carbon dots has been reported by other authors. Romero et al. [99] evaluated the use of different natural precursors for carbon dots synthesis for the detection of periodate anions in wastewater. In their study, higher fluorescence intensity (approximately 900 RFU) was observed in carbon dots from cruciferous vegetables [99]. Similarly, carbon dots with similar fluorescence intensity have been used for the detection of H_2_O_2_ and antioxidants [100]. These results suggest that carbon dots synthesized from waste tires could be used for sensing applications, although the full characterization of the synthesized carbon dots is the subject of further studies.

## 4. Conclusions

Pyrolytic carbon black’s properties strongly depend on the activating agent. H_2_SO_4_ leads to an impurity-free surface, removing the carbon black’s metal content and, therefore, improving its surface area with a micropore formation, while KOH increased its oxygen content, resulting in better stability in water suspension. A clear effect of KOH activation on ζ potential was observed, presenting a low absolute value when KOH activation was not involved in the process, regardless of the presence of H_2_SO_4_ activation. However, when KOH activation was performed, the absolute ζ potential value increased. This represents an additional benefit, since many applications of carbon black require the formation of stable aqueous dispersions. Pyrolytic carbon black was negatively charged with a porous structure; therefore, the exploration of applications such as metal adsorbents could be convenient. However, in this study, it was demonstrated that the pyrolytic carbon black could also be used as a precursor for carbon dots’ synthesis. The synthetized carbon dots presented a higher intensity in fluorescence emission at the range of 590 ± 35 nm and in the fraction from 0–10 kDa.

The results presented in this study demonstrate the feasibility of synthetizing carbon dots using waste tires as a precursor. Pyrolytic carbon black from waste tires resulted in being an excellent candidate for a nanomaterials precursor because of its high carbon content with the presence of some metal impurities enhancing graphitization, in addition to their narrow size distribution. Its use as a precursor not only reduces the process cost and consumption of non-renewable resources, but also provides alternatives to waste tire management. In this manner, a type of problematic waste can be converted into a carbon nanomaterial with potential applications in solar energy, energy storage, sensing applications, and other areas. Preliminary analysis of the carbon dots showed fluorescence intensity and size distribution results comparable to other carbon dots with successful sensing applications. Moreover, differences in their fluorescence were observed, and were associated with a higher oxidation degree caused by the activations performed on the pyrolytic carbon black. This provides a good starting point for further research, as it might provide information useful for understanding the origin of the properties of carbon dots—a subject that is still under debate. In this study, the recovery of carbon black from waste tires was performed by pyrolysis; however, further studies should investigate the performance of the solid fraction from the acid-activated waste tire obtained by wet pyrolysis, since this allows the elimination of the pre-drying requirement of the wet waste and a higher oxidation degree could be achieved.

## Figures and Tables

**Table 1:** Proximal and elemental analysis results from waste tire used as feedstock.

Analysis	Waste Tire			Tire Activated with H_2_SO_4_			Tire Activated with KOH			Tire Activated with H_2_SO_4_ and KOH		
	Mean	±	SD ^1^	Mean	±	SD	Mean	±	SD	Mean	±	SD
Moisture (%)	0.89	±	0.01	3.06	±	0.17	0.71	±	0.00	8.96	±	0.33
Ash (%)	9.99	±	0.52	7.20	±	0.51	9.75	±	0.41	3.16	±	0.29
Volatile matter (%)	66.95	±	3.60	48.36	±	0.49	66.07	±	1.35	38.87	±	0.16
Fixed carbon ^2^ (%)	22.16	±	4.14	41.38	±	0.83	23.47	±	1.77	49.02	±	0.46
Nitrogen (%)	1.59	±	0.08	1.63	±	0.08	1.57	±	0.18	1.45	±	0.01
Carbon (%)	82.25	±	1.29	72.90	±	0.13	81.49	±	0.29	61.43	±	0.15
Hydrogen (%)	7.34	±	0.09	4.99	±	0.00	7.14	±	0.04	4.26	±	0.06
Sulfur (%)	1.72	±	0.05	5.18	±	0.03	1.77	±	0.04	4.24	±	0.01
Oxygen (%)	2.21	±	0.15	9.74	±	0.04	3.74	±	0.02	14.90	±	4.42

^1^ SD: one standard deviation; ^2^ calculated by difference.

**Table 2:** Elemental analysis and metal content results of pyrolytic carbon black samples.

	CB			CB.H_2_SO_4_			CB.KOH			CB.H_2_SO_4_ + KOH		
	Mean	±	SD	Mean	±	SD	Mean	±	SD	Mean	±	SD
Nitrogen (%)	0.90	±	0.01	0.86	±	0.15	0.97	±	0.02	1.02	±	0.02
Carbon (%)	79.46	±	0.34	86.90	±	1.08	70.38	±	1.49	60.75	±	0.94
Hydrogen (%)	0.30	±	0.01	0.41	±	0.05	0.44	±	0.08	0.75	±	0.02
Sulfur (%)	3.09	±	0.06	1.36	±	0.02	3.04	±	0.04	4.45	±	0.06
Oxygen (%)	0.70	±	0.01	0.97	±	0.06	8.79	±	0.05	16.06	±	0.39
Cd (mg/kg)	0.00	±	0.01	0.30	±	0.08	0.45	±	0.21	0.11	±	0.00
Cr (mg/kg)	8.83	±	0.25	6.41	±	0.15	9.29	±	0.51	5.77	±	0.30
Fe (mg/kg)	862.47	±	2.50	147.93	±	1.14	549.67	±	2.15	80.98	±	0.61
Ni (mg/kg)	7.36	±	0.11	6.87	±	0.06	3.74	±	0.11	2.99	±	0.14
Pb (mg/kg)	4.77	±	0.18	1.99	±	0.30	6.73	±	0.23	3.16	±	0.19
Zn (mg/kg)	37543.33	±	565.89	1990.67	±	4.51	8032.67	±	74.90	158.13	±	0.72

CB: pyrolytic carbon black; CB.H_2_SO_4_: carbon black from H_2_SO_4_ activation; CB.KOH: carbon black from KOH activation; CB.H_2_SO_4_ + KOH: carbon black from H_2_SO_4_ and KOH activation. See text for details.

**Table 3:** Fitting parameters from Raman spectra of pyrolytic carbon black samples.

Fitting Parameters	CB	CB.H_2_SO_4_	CB.KOH	CB.H_2_SO_4_ + KOH
I_D_/I_G_	0.96	1.01	0.94	0.93
D band position (cm^−1^)	1345	1345	1361	1360
G band position (cm^−1^)	1584	1579	1577	1574
D band height	0.79	0.84	0.70	0.37
G band height	0.82	0.83	0.74	0.39

**Table 4:** ζ potential results of carbon black samples.

Sample	ζ Potential (mV)			
	Mean	±	SD	
CB	−23.88	±	1.20	A
CB.H_2_SO_4_	−23.75	±	2.15	A
CB.KOH	−37.35	±	1.42	
CB.H_2_SO_4_ + KOH	−46.94	±	1.90	

Mean values with the same letter are not statistically different (Tukey’s honestly significant difference (HSD), *p*-value > 0.05).
